# Latent dirichlet allocation for double clustering (LDA-DC): discovering patients phenotypes and cell populations within a single Bayesian framework

**DOI:** 10.1186/s12859-023-05177-4

**Published:** 2023-02-23

**Authors:** Elie-Julien El Hachem, Nataliya Sokolovska, Hedi Soula

**Affiliations:** Sorbonne University, INSERM, Nutrition and Obesities: Systemic Approaches, NutriOmique, 91 Boulevard de l’hôpital, 75013 Paris, France

**Keywords:** Double clustering, Bayesian topic modelling, Latent Dirichlet allocation, Precision medicine

## Abstract

**Background:**

Current clinical routines rely more and more on “omics” data such as flow cytometry data from host and microbiota. Cohorts variability in addition to patients’ heterogeneity and huge dimensions make it difficult to understand underlying structure of the data and decipher pathologies. Patients stratification and diagnostics from such complex data are extremely challenging. There is an acute need to develop novel statistical machine learning methods that are robust with respect to the data heterogeneity, efficient from the computational viewpoint, and can be understood by human experts.

**Results:**

We propose a novel approach to stratify cell-based observations within a single probabilistic framework, i.e., to extract meaningful phenotypes from both patients and cells data simultaneously. We define this problem as a double clustering problem that we tackle with the proposed approach. Our method is a practical extension of the Latent Dirichlet Allocation and is used for the Double Clustering task (LDA-DC). We first validate the method on artificial datasets, then we apply our method to two real problems of patients stratification based on cytometry and microbiota data. We observe that the LDA-DC returns clusters of patients and also clusters of cells related to patients’ conditions. We also construct a graphical representation of the results that can be easily understood by humans and are, therefore, of a big help for experts involved in pre-clinical research.

**Supplementary Information:**

The online version contains supplementary material available at 10.1186/s12859-023-05177-4.

## Background

Human disorders have a highly multifactorial nature and depend on genetic, behavioral, socio-economic, and environmental factors. There are many examples of such complex diseases: cardiovascular diseases, non-alcoholic liver cirrhosis, type II diabetes, or even other pathologies such as autoimmune diseases [1] to name a few. The number of subjects with metabolic diseases, cancers, and autoimmune pathologies has increased significantly in recent years, making research in this field a public health priority [2].

In parallel, bioclinical routine datasets have expanded in conjunction with all kind of “omics” data, from both the host and microbiota, as well as metabolomic, proteomic, and cytometry data [3]. All these types of data have some underlying structure on their own, taking values on different scales, with different variability, and are differently distributed. In addition, human patients are an equally important source of variability even among carefully selected cohorts: phenotypic variability (age, gender, previous conditions), dietary habits, bad versus good responders to treatment, etc. As a result, the amount of available heterogeneous data has increased exponentially. In particular, cell based techniques such as single cell RNA sequencing (scRNA-seq) revolutionized the field of life sciences by bringing an unprecedented resolution to study heterogeneity in cell populations [4]. So, single-cell transcriptome profiling of pathologic tissue isolates allows the characterization of heterogeneous pathologic cells along with neighboring immune cells. More precisely, flow cytometry and scRNAseq are cell-level data describing heterogeneous cells’ behavior. The most recent results, either take into account the cell heterogeneity by itself (e.g., by deriving cell lineage) or compress the information into population proportion after a (usually arbitrary) clustering for patient-to-patient analyses that prevents us from simplistic data fusion in order to extract meaningful information.

Flow cytometry workflow, e.g., computes a so-called gating where bi-axial plots are used by human experts to distinct cells. This method is often performed by a researcher and is, therefore, accurate but expensive. A more computationally efficient way to identify cell populations are machine learning clustering methods. Among the state-of-the-art clustering methods for scRNA-seq data for cell-type identification are distance-based partitioning, density-based clustering, or graph-based clustering methods [5–8]. One of the most widely used exploration method for cell data is the t-SNE [9] which is a probabilistic dimensionality reduction and visualization method. It is not only widely used in the single cell analysis but also a number of methods were developed based on the t-SNE. So, in ACCENSE [10] and ClusterX [11], the t-SNE is used to estimate the density and also to project the data before the cell populations are identified. Another approach, viSNE [12], where each cell is a point in high-dimensional space, proposes a distributed implementation of the t-SNE. Different combinations of t-SNE and graphical methods were explored, e.g., PhenoGraph [13], where a nearest-neighbor graph is applied to cell data to reveal the partitioning, determines phenotypes in single cell data. A similar idea is also considered in Xshift [14]: the *k*-nearest-neighbor algorithm is used to identify connectivity and density peaks in cell data.

Dimensionality reduction is a natural way to process the single cell data. So, FlowSOM [15] is a cell clustering technique based on Self-Organising Maps (SOM), where the result of stratification is a grid of cell clusters, and it can be visualized by showing the average marker values of each identified cluster. Some practical packages, e.g., CITRUS [16] which relies on hierarchical clustering, were proposed. Their goal is to apply some standard robust clustering methods to the single cell data.

Currently, research is focused on the development of graph-based clustering methods. Indeed, in [17], the authors compare different graph clustering methods for community identification. These methods can take into account a single network (i.e., co-expression, protein-protein interaction) or aggregate information of several networks. Among the most efficient methods are kernel clustering, modularity optimization, random-walk-based methods and local methods allowing to identify communities related to particular pathologies. In parallel, [18] have developed a layer specific module in multi-layer network based on non-negative matrix factorization (LSNMF). In this approach, LSNMF learns latent features of vertices and decomposes them into two types of features: common and specific ones, where the specificity of features for vertices is explicitly measured, thereby improving the accuracy of algorithms. As a result of different experiments, the features identified in these modules appeared to accurately characterize different modules. Moreover, the attention of the community has been extended to the clustering of scRNA-seq data, through the use of network-based methods. Indeed, [19] has developed a network-based structural learning non-negative matrix factorization algorithm (SLNMF) for cell type identification. The authors show that their approach based on the topology of the reconstructed from data network, is much more efficient and accurate for cell types identification than standard approaches based on expression data.

Recently, the attention of the systems biology community was drawn by Bayesian probabilistic methods. The intuition behind these approaches in relation to the biological tasks is to model individuals who belong to multiple populations. For example, [20] proposes a method based on a Dirichlet mixture model to cluster single cell transcriptomic data, pointing out that model-based (probabilistic) methods are underexplored for single cell data analysis. The estimation of the model is done using the Expectation-Maximisation (EM) algorithm.

Some attempts to adopt the Latent Dirichlet Allocation (LDA) to the single cell data were recently made. So, [21] applied the LDA to a database with approximately 50 human tissues to discover similarities between them; the LDA was also tested on single cell mouse data to discover variations in early embryonic development stages. An important characteristic of the single-cell data is that the data is structured; [22] states that any clustering method for the single-cell data should account for the hierarchical structure of cell types, and proposes new metrics to evaluate clustering performance. To construct tree structures which reflect the hierarchical nature of single cell data, [23] explore a hierarchical extension of the LDA to identify clusters of cells. Cellular LDA (Celda) was introduced by [24] to perform bi-clustering of co-expressed genes and also of cells into subpopulations. The Celda takes into account the hierarchical relationships in data.

Recently, the Latent Dirichlet Allocation (LDA) was considered to partition the single-cell data [25, 26]: the LDA was applied to binary data, where each cell was treated as a document, and each chromatin site (chromatic contact) was considered as a word. So, [25] proposed a Bayesian topic modeling framework called cisTopic for robust identification of cell types. In [26], the LDA is tested on the extremely sparse data to capture cell type differences.

From the analytical viewpoint, the single cell data are huge-dimensional matrices produced for each subject. The data dimension, i.e., the number of cells, vary from one individual to another, and note that cell types, as well as the correspondence between the cell populations of the subjects, has to be identified before applying any statistical machine learning method. We refer to the challenge we introduce and consider here as to a *double clustering* problem, where the aim is to simultaneously, purely from observations without any prior knowledge determine cell types, as well as stratify patients in order to study mechanisms of pathologies explained by particular cell subpopulations.

In this contribution, we propose the *Latent Dirichlet Allocation for Double Clustering* (LDA-DC) which is a novel method to identify cell types from flow cytometry data, and cluster patients in the same flow. We discuss the advantages coming from the Bayesian probabilistic nature of our approach, and we illustrate its strengths on real benchmarks.

## Methods

### Latent Dirichlet allocation for double clustering

**Fig. 1:**
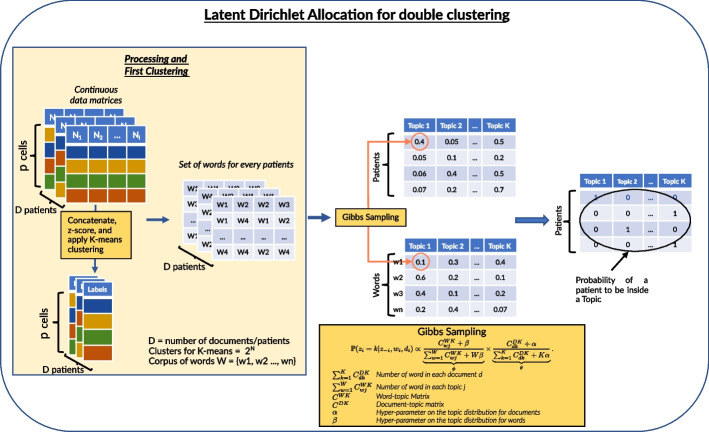
The proposed pipeline to perform the double clustering: from the observations (where one patient is represented by a matrix) to the conditional probability distributions of clusters given patients and cellular types given clusters. *N* is the number of fluorescent markers, *K* is the number of clusters, *W* is the number of words

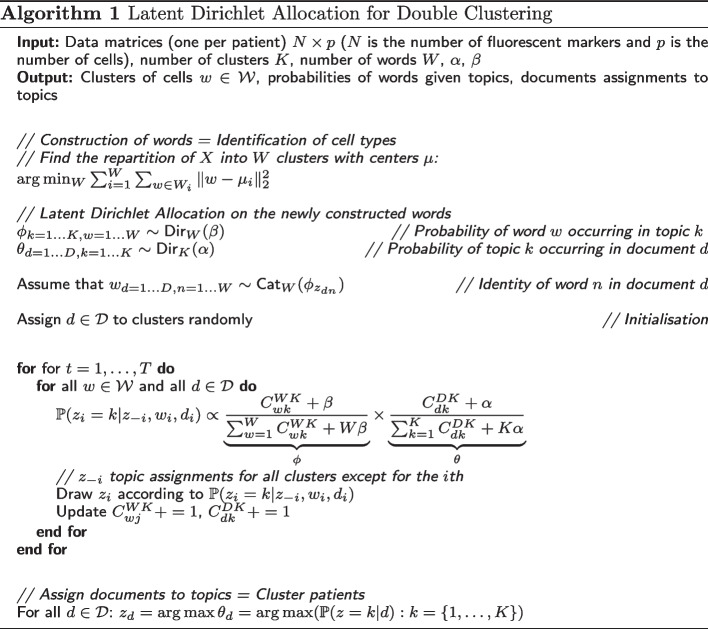



Latent Dirichlet Allocation (LDA) [27] was originally proposed as a probabilistic topic modeling method. It is a Bayesian approach which was developed to identify topics given a corpus of documents, where the topics are not known in advance. Note that the standard LDA considers discrete (counts) data. The LDA is based on several assumptions. First, each document can be represented by a mixture of topics (Fig. 1). Second, as a result of the learning procedure, one learns not only the topic distribution representing each document, but also the distribution of words associated with each topic. The word distribution is helpful to interpret the topics. The main goal of the LDA learning procedure is to estimate the model parameters $\theta$ (words distribution describing topics) and $\phi$ (topics distribution describing documents). The Latent Dirichlet Allocation framework is formalised as follows. The topics are distributed according to a Dirichlet distribution:

1 $$\begin{aligned} \theta \sim \text {Dirichlet}(\alpha ), \end{aligned}$$

where $\alpha$ is a hyper-parameter. The distribution of words is also modeled by the Dirichlet:

2 $$\begin{aligned} \phi \sim \text {Dirichlet}(\beta ), \end{aligned}$$

where $\beta$ is another hyper-parameter of the LDA model to control the topic-words distribution.

To estimate the parameters of the model and to perform clustering, we are particularly interested in the following conditional probability computed from two Dirichlet distributions. The conditional probability of assigning *i*th token to cluster *j* is given:

3 $$\begin{aligned} {{\mathbb {P}}(z_i = j | z_{-i}, w_i, d_i) \propto \underbrace{\frac{C_{wj}^{WK} + \beta }{\sum _{w=1}^W C_{wj}^{WK} + W \beta }}_{\phi } \times \underbrace{\frac{C_{dk}^{DK} + \alpha }{\sum _{k=1}^K C_{dk}^{DK} + K \alpha }}_{\theta }}, \end{aligned}$$

where *D* is the number of documents, *W* is the number of words, *K* is the number of clusters (topics), $C^{WK}$ is the word-topic matrix, $\sum _{w=1}^W C_{wj}^{WK}$ is the total number of words in each topic, $C^{DK}$ is the document-topic matrix, $C_{dk}^{DK}$ is the total number of words in a document; $z_{-i}$ is the topic assignments for all other topics.

The intuition behind the hyper-parameters is as follows. The higher $\alpha$, the more likely a document is described by more topics. The higher $\beta$, the more likely each topic is described by more words. As in the majority of clustering methods, the number of topics (clusters) has to be fixed.

Although a number of optimization approaches were proposed to estimate the parameters of the LDA framework, we use the standard Gibbs sampling [28] in our numerical experiments.

The originality of our approach is the extension of the LDA to the double clustering framework. The complete learning procedure, called *Latent Dirichlet Allocation for Double Clustering (LDA-DC)* is drafted as Algorithm 1. The algorithm takes the patients data matrices, where the number of lines *p* is the number of cells, and *N* is the number of columns (fluorescence markers) (Fig. 1). Note that cells are different across patients, and a straightforward application of any state-of-the-art machine learning method such as Support Vector Machines or Random Forests, is not possible. The first step of the double clustering is the identification of the cell types. Using the (topic modeling) LDA terminology, the cell identification is the identification of words (note that in the standard LDA the words are well-defined and provided).

Taking into consideration that our algorithm was developed with the single cell data in mind, where the form of the distribution is supposed to be known, i.e., Gaussian, the words (cell types) identification is done using the *K*-means clustering which is known to be more robust compared to the Expectation-Maximisation algorithm that is sensitive to its initialization.

Once the cell types are fixed, the LDA can be efficiently used to estimate both the probabilities of a phenotype given a patient, and the probability of a cell type given a phenotype. Thus in addition to provide a topic for each patient, our method provides a topic for each cell phenotype.

### Simulated data

*Cell generation:* We constructed an artificial dataset to validate the proposed method. In order to mimic real flow cytometry datasets, our main hypothesis for the data generation is that the underlying distribution of fluorescent data can be efficiently approximated by (multivariate) Gaussian distributions. So, each marker can be seen as a mixture of two Gaussians with different means: one is associated with positive subsets (high mean), and the second one is associated with negative subsets (low mean). In order to test for robustness we can vary the standard deviation (std) of the distribution with high standard deviation making more difficult to separate low from high. Therefore, the phenotype of a cell is a real vector of dimension *N* (think of *N* as the number of fluorescent marker under consideration). To these continuous vectors we have associated the binary vector of dimension *N* with highs and lows describing the cell’s phenotype. There are therefore $2^N$ possible cell phenotypes.

*Simulated patients:* In order to create patients, we construct probability distribution vectors of cell type density that differ according to patients’ phenotype, i.e., different classes of patients will have different cells’ type distribution. Here, we tested two cases: two classes of patients and four classes of patients with prescribed cell’s type distribution for these 2 or 4 classes. We can simulate the patients by choosing their cell’s type distribution and compute the cell’s fluorescent values according to the current cell type. Note that at this point we also fix the standard deviation. Thus, an artificial patient is represented by a random subset of cells whose number is $p_{cell}$ from which we derive the cell type according to the phenotype distribution. We can compute real values for the cell according to its type. The distributions are chosen using a simple parameter that can vary the distance between classes.

### Real benchmarks

To illustrate the efficiency of the proposed method, we selected two real annotated benchmarks.

*AML (Acute Myeloid Leukemia) dataset:* This dataset [29] has 2872 samples of flow cytometry standards collected from 359 AML (n=43) and non-AML (n=316) individuals. It contains results from 8 experiments corresponding to different tubes with different markers (note that tube 1 is an isotope control, and tube 8 is unstained).

*Cytometry and genus data* The dataset we use contains FACS cytometry and 16rRNA sequencing data coming from two studies: [30] and [31] respectively. Note that the original cytometry data comes from [31] and are paired with the 16rRNA data. However, we use the data from [30], since this data set is pre-processed (noise reduction using various transformations and algorithmic methods, see Analysis part in [30] for more details). So, we have a cohort composed of patients diagnosed with Crohn’s disease (CD, $n=29$) and healthy subjects (HC, $n=66$). The cohort of patients having the Crohn’s disease is described in details in [32], and the samples of HC patients come from the Flemish Gut Flora Project [33].

### The double clustering workflow

Here we provide the details of the proposed approach. We discuss its application to our artificial dataset, where we generate both, cells populations and patients in a controlled manner and compare it with the ground truth for both the cell’s type and patient’s phenotype. For the subsequent real datasets the method is strictly identical.

*Cells clustering:* We generate according to the method described above about 50 subjects per phenotype (note that cell type density distribution is a vector of size $2^N$). As already mentioned, we focus on a setting with 2 and with 4 phenotypes; 50 patients per phenotype. Per one patient, we generate a matrix of $10^4$ cells measurements. These measurements can be encoded as continuous and binary (low/high) values. We perform the following pre-processing: we concatenate all patients and apply the *Z*-score on all patients. Then, we apply a *K*-means on the concatenated observations. Note that the number of clusters (cells types) is fixed to $2^N$. As a result of the cells clustering, each cell (of each patient) is assigned to a cluster, and we can consider the counts of cells in each cluster.

*Patients clustering:* In the previous step (cells clustering), we obtained the matrix of cells types counts per patient, where a cell type corresponds to the class assigned to the cell by the clustering method. The Latent Dirichlet Allocation (LDA) can be directly applied to the count matrix. Using the topic modeling terminology, we can imagine that the patients are considered as documents, and words are considered as cell types. The LDA model gives us the probability for each patient to be assigned to each cluster. Note that the number of phenotypes is also fixed in advance. The resulting conditional probability can be used in various ways. Traditionally, it is used to cluster observations based on the maximal probability value. Alternatively, we can apply a hierarchical clustering, e.g., with a tree cut to visualize and to explore the results.

## Results

### Validation of the proposed method on simulated data

**Fig. 2:**
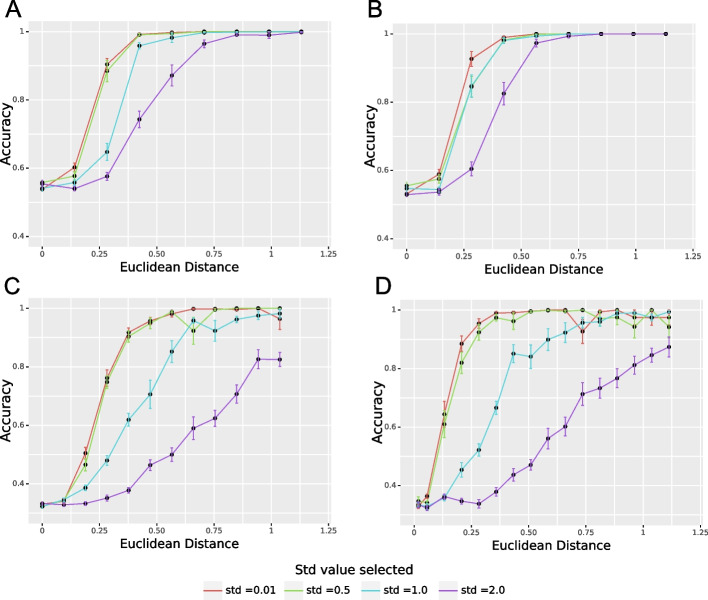
Mean accuracy for the simulated scenarios with 2 and 4 phenotypes. We vary the distance between the phenotype vectors, standard deviation (std), and the number of clusters (*k*), $n_{cell} = 10 000$. **A** Mean accuracy for 2 phenotypes, $N = 2$, $W = 4$. **B** Mean accuracy for 2 phenotypes, $N = 4$, $W = 16$. **C** Mean accuracy for 4 phenotypes, $N=2$, $W = 4$. **D** Mean accuracy for 4 phenotypes, $N=4$, $W = 16$

We tested the double clustering approach on different scenarios. We tested 2 and 4 phenotypes, and we varied the distance between the probability vectors to vary the difficulty of the clustering problem. If the Euclidean distance between the phenotype probability vectors is small, the clusters are not well-separable, there is a significant overlap between the groups. If the Euclidean distance between the probability vectors associated with the phenotypes is big, the clusters are easily separable, and we can expect a reasonable performance. The overlap between the clusters can be controlled by the variance.

Figure 2 illustrates the results on the synthetic dataset. The subplots A and B show our results for the case with 2 phenotypes, andC and D illustrate the setting with 4 phenotypes. The subplots A and C report the results for the problem with a lower dimension (4), and the subplots B and D show the accuracy for the case with more features (16). Thus, when two groups of patients (Fig. 2A and B) have the phenotype probability vectors that are difficult to distinguish (Euclidean distance is lower than 0.25 in the experiments), the accuracy is close to 50%, which is what is expected. If the number of dimensions increases (the size of vocabulary in the LDA increases), the accuracy does not seem to degrade (Fig. 2A and B). We obtain similar results for the setting with 4 phenotypes (Fig. 2C and D). If the clusters are hardly separable (generated with a Euclidean distance lower than 0.25), the accuracy is close to 30%. Increasing the dimensionality of the problem does not alter the performance significantly.

### Real high-dimensional datasets

**Fig. 3:**
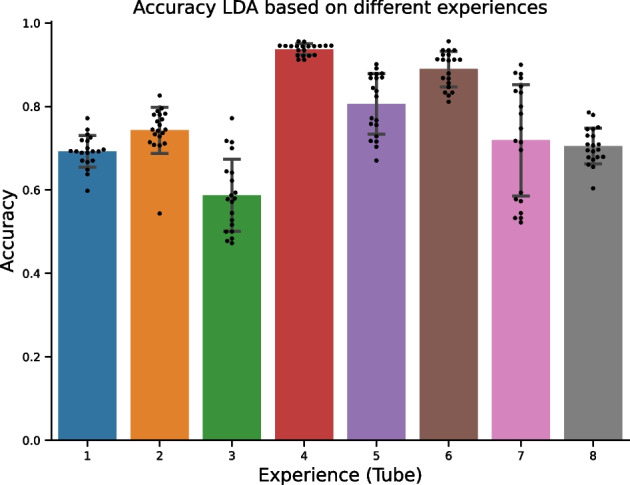
The accuracy of the double clustering method on the 8 tubes of the AML dataset

#### Acute myeloid leukemia: AML dataset

The AML benchmark dataset is an unbalanced dataset of individuals with AML syndrome and healthy subjects. This dataset includes flow cytometry measurements for several batches (tubes) of different biomarkers sets. In order to balance the learning procedure, for each tube, we selected the same number of AML and non-AML patients excluding several non-AML patients. First, we applied the *K*-means with $2^{D}$ clusters, where *D* is the number of markers in a tube. Then, we annotate each cell according to its origin (patient) and its cluster. For each patient, we obtain a list of cell types. We also define the vocabulary equivalent to the number of clusters of the *K*-means as well as the number of topics (here 2, since there are 2 conditions: AML and non-AML). At this point, each patient (observation) is a list of cell types, and we apply the LDA on the count data. In each experiment corresponding to each tube, we assign each patient to a cluster. We have the ground truth for all observations, however, since we consider a clustering task, we face the label switching problem. So, to cope with the label switching problem, we applied the majority vote to the obtained clusters. We compared the final clustering to the real classes (AML and non-AML). The accuracy is shown on Fig. 3. Note that some tubes are more predictive than others, due to different biomarkers used in the experiments.

We run a cross validation (number of folds is equal to 20 in our experiments). It is important to perform the cross-validation here, since the number of ill and healthy individuals is unbalanced, at each run we sample (uniformly) the same number of observations from both classes.

To compare with a baseline approach, we tested the standard *K*-means instead of the LDA approach to identify the clusters of subjects, and we found out that there is not any advantage in terms of predictive performance of the *K*-means over the LDA. We observed that our method is less efficient on the data of tube 6 (adjusted p-value $=$ 0.002 for tube 4 and adjusted p-value $=$ 0.001 for tube 6; the results of the t-test and false discovery rate adjustment are provided in Additional file 1: Supplementary Informations Table S1). However, the LDA-DC provides us with some additional information. Indeed, we are able to extract the probability distribution of cell types related to each phenotype. So, we can detect which cell phenotypes drive the clustering and explain the disease.

**Fig. 4:**
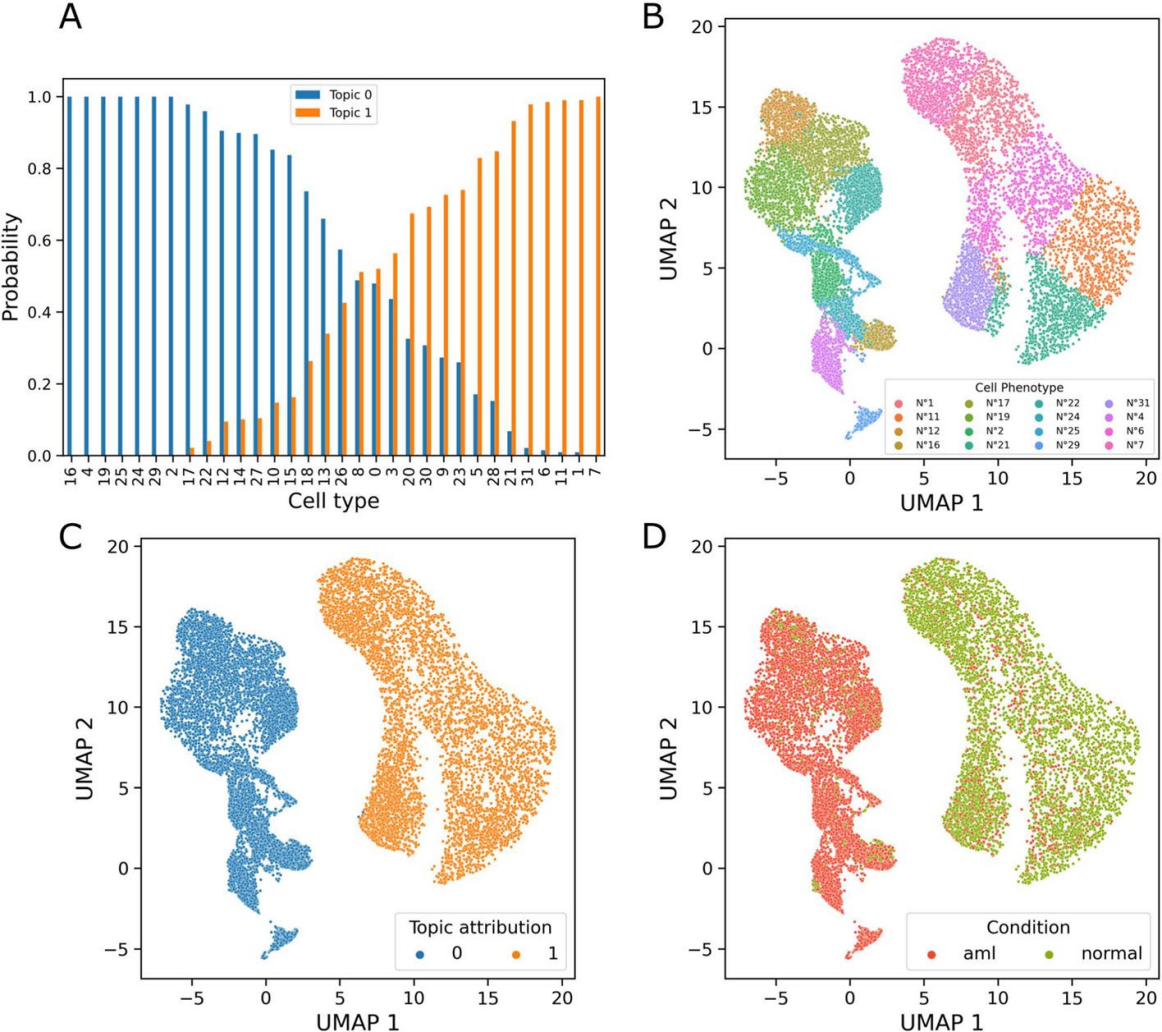
**A** Conditional probabilities of cell types given the topics. **B** Cells projection (UMAP), the cells plotted are ones whose probability of assignment to the clusters is bigger than 90%; **C** the cells are colored according to the topic (ill/healthy); **D** the cells are colored according to the true label (phenotype)

Figure 4 illustrates our findings obtained with the LDA-DC. Panel A shows the topic density for each cell type. Although some cells are present in both, it is clear that some cell populations are associated with high probability, with the patient’s phenotypes. Visualizing the cells with the Uniform Manifold Approximation and Projection for Dimension Reduction (UMAP) [34] methods (panels B, C, D), we color-coded the cells according to: (1) appearance in a cell population (subplot B), (2) cells associated with disease/healthy estimated clusters (subplot C), and (3) cells colored according to the true clinical condition (panel D). In the last panel, we quantified the number of cells assigned to Topics 0 and 1 from AML and non-AML patients (see Additional file 1: Figure S1 and Additional file 1: Table S2), and tested the differences using Chi-squared test. We found out that the p-value < 2.2e$-$16, indicating that there is a significant difference in the distribution of the cells between AML and non-AML individuals within the two topics. Thus, the last panel is an indication that the cell populations we found are clearly associated with the disease. Our numerical results confirm that the double clustering predicts the clinical conditions with the unsupervised method and provides new information which allow us to relate the disease (or its absence) with cell subpopulations.

#### Cytometry and genus data: Crohn Disease Prediction

Nowadays, the number of studies dedicated to the human microbiota, increases steadily. We focused on the problem described in [31], where there two phenotypes: Crohn disease (CD) and healthy subjects. Our goal is to apply the LDA-DC to stratify the patients efficiently based on their cytometry as well as sequencing data. First, we selected all the data coming from [30] and selected 4 markers identified in the paper as markers of membrane bacteria. We tested a setting with two topics to separate the patients into two groups. However, the markers used were not adapted and we could not stratify the patients into two groups correctly. We arbitrarily fixed the number of topics to 8 (we also tested several values using the grid search), and applied our double clustering workflow.

**Fig. 5:**
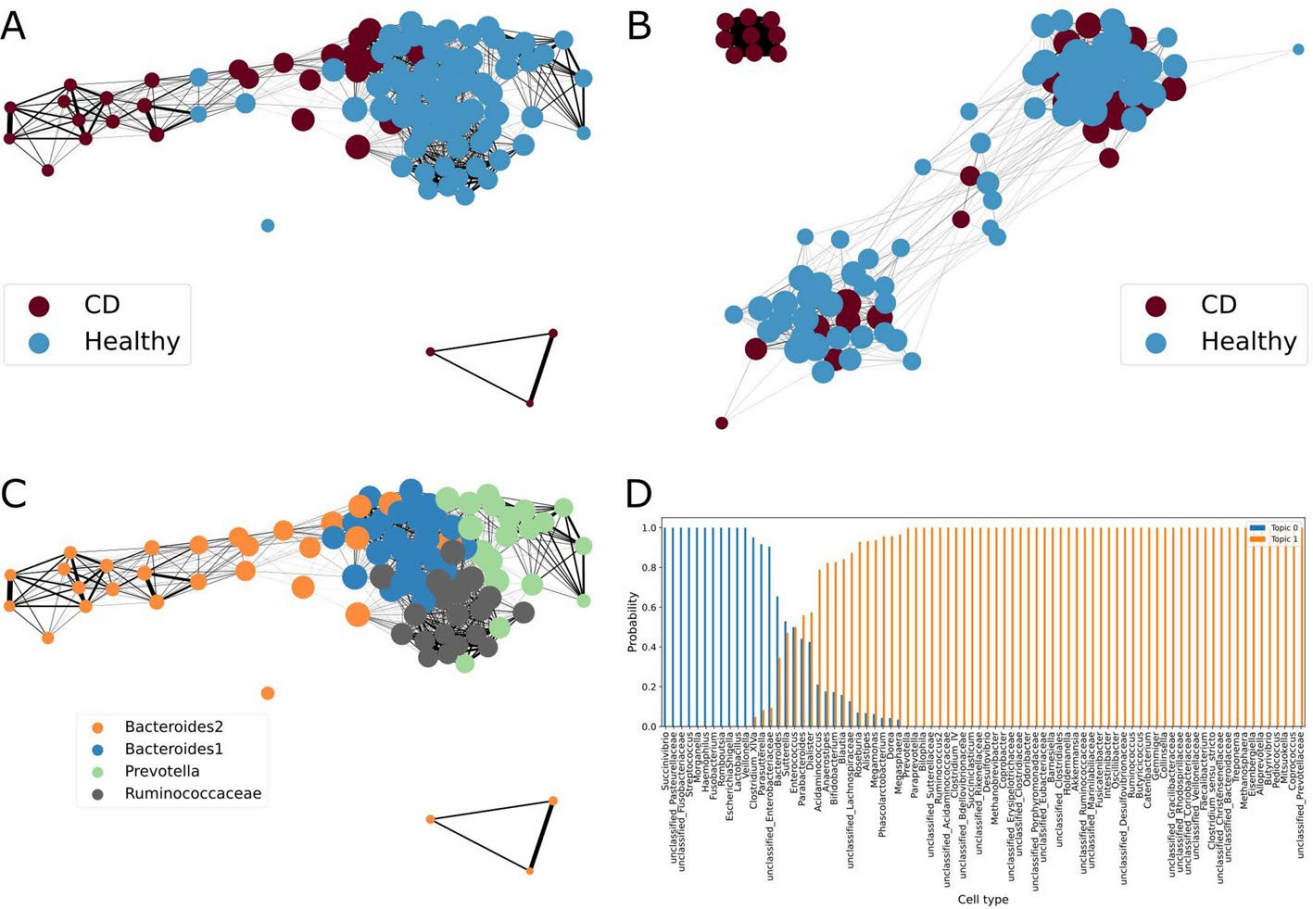
**A** Networks of patients constructed from 40 LDA-DC runs from the genus data; **B** Networks from the cytometry data; **C** Network of patients based on the genus data, where the colour of the node represents patients enterotypes; **D** Conditional probability distributions of bacteria given a cluster (here 2 clusters considered: CD and Healthy)

To stratify the patients and see whether the result is consistent, we repeated the training procedure 40 times, and we count the number of times each patient is associated with a particular cluster. We considered a diagonal matrix, where patients are in columns and rows, showing how many times the patients are clustered together, and we normalized these values by the number of experiments. Also, we applied an arbitrary threshold of 30%, and removed the links between patients that occur less than 30% of experiments. This matrix can be considered as one describing connectivity in the data, and we visualise a network where nodes are patients, edges are the connections between the patients, and edge weights are the connection frequencies. The obtained networks are shown on Fig. 5. Adding more information to such a graph, we modify the size of the nodes so that it reflects the connectivity (the bigger node degree, the bigger the node). The patients are colored according to their clinical condition (CD or Healthy), or to their enterotypes which are also provided with the dataset.

So, a network generated from the cytometry data, can separate patients into 3 groups: one group with data of sick patients only, and two other groups containing both sick and healthy subjects (Fig. 5B). However, one of these mixed groups contains significantly more ill patients.

Applying the same approach to the genus data, we observe that patients are well clustered by the double clustering method. On the left (orange nodes) we have a cluster of mainly sick patients, and on the right healthy ones (Fig. 5A). Then we consider the patients enterotypes (provided with the data and identified by [31]), and we notice that the patients from left to right form a continuum of enterotypes; and are all well separated too.

Subsequently, we decided to set the number of clusters—as done previously—(topics in the LDA terminology) to 2, and to stratify patients into two groups according to their conditions. Our aim here is to identify bacteria related to the disease. Indeed, we identified some bacteria that are linked to specific cell phenotypes and to conditions. Thus, we are able to find bacteria related to Crohn disease, and by extension, cells that drive the phenotypes. They are shown on Fig. 5, subplot D.

## Discussion

In this article, we proposed a simple method to obtain clusters of cells and patients simultaneously in the context of bioclinical datasets such as flow cytometry. We first validated our approach on simulated data: we noticed that it separates patients with a reasonable accuracy which depends on the difficulty of the clustering problem. We also showed how robust this method is according to noise (embodied as the standard deviation of the Gaussian). This result is a simple proof of concept of the method for cytometry-like data.

We then applied our approach on two publicly available datasets: For the AML study, our method correctly predicted patients status but also provided cell phenotypes associated to this status. Indeed, we isolated some cellular phenotypes associated with AML. This phenotypes are identified by specifics fluorescence values and biological markers that can be investigated further. Therefore, one of our main results is shown on Fig. 4, panel D: the identified cell populations are well distinguished according to the clinical condition (AML versus non-AML).

Finally, we applied our approach to microbiota data where we have access to two data types: cytometry data and bacterial abundance. The cytometry dataset is mainly targeting bacterial membrane proteins. Wehereas, the second one is a count matrix for each patient, where each bacteria is identified (genus data). Our main result on the cytometry dataset is the patients’ stratification according to their clinical status using two topics, the separation is reasonable but not perfect. The error rate in this experiment can be explained either by the heterogeneity of bacteria in their membrane protein composition, or by the fact that the type of targeted membrane proteins are not specific to one type or sub-types of bacteria and are, therefore, not very good predictors of the Crohn disease. Even if these markers are not strong predictors, the clustering results are still reasonable (accuracy $\approx 70\%$). On the other hand, using the genus data, we are able to separate correctly the patients into two distinct groups. As for the AML experiement, we were able to pinpoint actual bacteria species/genus directly associated with Crohn disease and these can be further investigated.

Indeed, on Fig. 5 it is easy to see the patients partitioning based on their enterotypes (subplot C). On subplot D, we show the bacteria which are related to the disease. For cluster 0, which corresponds to the Crohn’s disease, we identified the following bacteria: *Fusobacteriacceae* whose abundance increases in Crohn’s disease [35–37], *Enterobacteriacean* and *Veillonella* reported by [35] to be increased with the Crohn’s disease. [37] also state that the abundance of *Haemophilus* increases with the Crohn’s condition. Topic 1 (Fig. 5, subplot D) is associated with the healthy individuals, and we identified different bacteria known to reflect the healthy condition. So, [38] states that *Faecalibacterium, Clostridium IV, Roseburia, Ruminococcus* are decreased in patients with the Crohn’s disease compared to healthy subjects. In addition, [37] states that *Blautia, Coprococcus* (identified in topic 1) are less abundant in Crohn’s patients compared to healthy subjects. In this regard, we confirm that bacteria identified in topic 0 are markers of the Crohn’s disease, while those identified in topic 1 are markers of the healthy condition.

## Conclusions

In this paper, we introduce a new method called Latent Dirichlet Allocation for Double Clustering (LDA-DC) to cluster features (e.g., cells) and patients from high dimensional data. Globally speaking, this method unifies clustering methods within one Bayesian framework to group cells into different cellular phenotypes from quantitative data, and stratify patients based on the clustered cells. We validated the method, and illustrated that it performs both, cells and patients partitioning reasonably well (we considered accuracy, since the ground truth was provided for the cohorts). This method allows us to stratify patients and cells simultaneously. In addition, it allows us to identify relationships between cells phenotypes and patients clusters. Thus, we obtain more information compared to the majority of the state-of-the-art clustering methods.

Currently we are working on a hierarchical version of the proposed LDA-DC which is in some sense similar to the hLDA actively used by the topic modeling community. A particular interest to develop this direction is a hierarchical nature of the cells data. Another avenue of research is to propose novel methods based on soft clustering of the cells: note that the Expectation-Maximization method can be considered as a baseline method only, due to its known drawbacks such as initialization and scalability issues. An important question to consider is also cost-sensitive clustering, since real data are often extremely unbalanced. We would also like to go further into the graphical representations of the results, since such a visual clustering showing more refined phenotypes could be an avenue for the development of methods of personalized medicine.

## Supplementary information

**Additional file 1. Table S1:** P-values and adjusted P-values of comparisons between K-means and LDA accuracies for the different AML experiment tubes. Adjusted p-value was performed using FDR methodology. Mean accuracy for multiple runs (20 runs) using LDA-DC method, and K-means for each tube. **Figure S1:** Quantification of cells assigned to Topic 0 and 1 from AML and non-AML patients associated to Figure 4D. The data are normalized by the number of sampled cells for AML and non-AML individuals. **Table S2:** Confusion Matrix associated to the heatmap Figure S1. We performed a χ^2^-test with p-value < 2.2e-16 indicating that there is a significant difference in the distribution of the cells from AML and normal patients within the two topics.

## Data Availability

Our code (Python) is publicly available for scientific purposes at: https://github.com/ElieElHachem/LDADC. AML data from [29] are available at https://flowrepository.org under the accession FR-FCM-ZZYA. Cytometry data from [30] are available at https://flowrepository.org under the accession FR-FCM-ZYVH, and 16sRNA (genus data) from [31] are stored on github at https://github.com/prubbens/PhenoGMM_CD.
