# Supplementary material for: Latent dirichlet allocation for double clustering (LDA-DC): discovering patients phenotypes and cell populations within a single Bayesian framework
Source: BMC Bioinformatics. 2023 Feb 23;24:61. doi: 10.1186/s12859-023-05177-4 (PMC9948385; doi:10.1186/s12859-023-05177-4)
Supplement: Supplementary file 1 — Additional file 1. Table S1: P-values and adjusted P-values of comparisons between K-means and LDA accuracies for the different AML experiment tubes. Adjusted p-value was performed using FDR methodology. Mean accuracy for multiple runs (20 runs) using LDA-DC method, and K-means for each tube. Figure S1: Quantification of cells assigned to Topic 0 and 1 from AML and non-AML patients associated to Figure 4D. The data are normalized by the number of sampled cells for AML and non-AML individuals. Table S2: Confusion Matrix associated to the heatmap Figure S1. We performed a χ2-test with p-value < 2.2e-16 indicating that there is a significant difference in the distribution of the cells from AML and normal patients within the two topics. [file 12859_2023_5177_MOESM1_ESM.pdf]

Latent Dirichlet Allocation for Double Clustering (LDA-DC): Discovering patients phenotypes and cell populations within a single Bayesian framework

January 2023

Supplementary Informations

Tube	p-value t-test	Adjusted p-value	Mean Accuracy LDA-DC	Mean Accuracy K-means
1	0.804170	0.804170	0.692333	0.695610
2	0.150241	0.300481	0.742695	0.721526
3	0.372407	0.595851	0.587136	0.569103
4	0.000402	0.001608	0.937044	0.954188
5	0.715108	0.804170	0.806294	0.796111
6	0.000131	0.001044	0.889357	0.936771
7	0.136290	0.300481	0.718888	0.661792
8	0.450157	0.600210	0.705092	0.690496

Table S1: P-values and adjusted P-values of comparisons between K-means and LDA accuracies for the different AML experiment tubes. Adjusted p-value was performed using FDR methodology. Mean accuracy for multiple runs (20 runs) using LDA-DC method, and K-means for each tube.

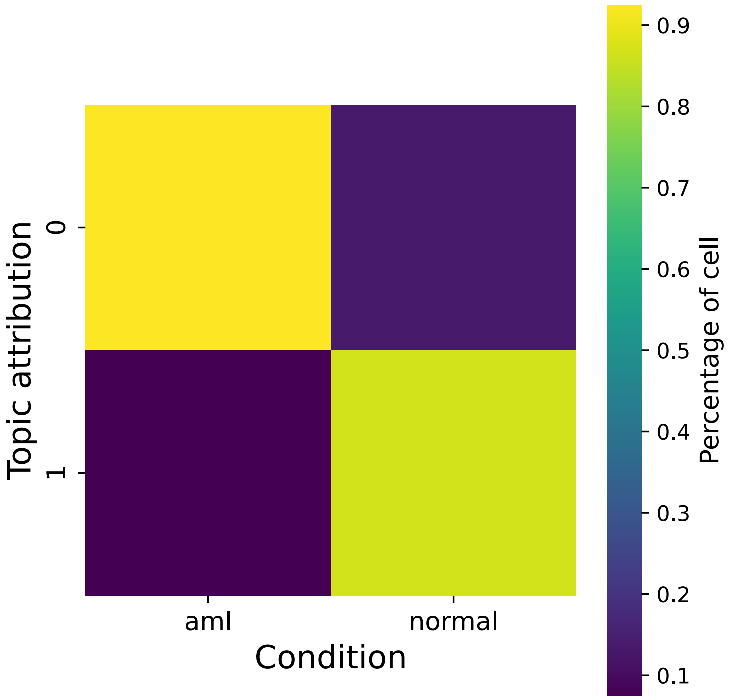

Figure S1: Quantification of cells assigned to Topic 0 and 1 from AML and non-AML patients associated to Figure 4D. The data are normalized by the number of sampled cells for AML and non-AML individuals.

Topic \ Condition	AML	Normal
0	0.924839	0.131614
1	0.075161	0.868386

Table S2: Confusion Matrix associated to the heatmap Figure S1. We performed a χ^2 -test with p-value < 2.2e-16 indicating that there is a significant difference in the distribution of the cells from AML and normal patients within the two topics.
